# The Use of the Opsonic Index in the Diagnosis of Tuberculosis

**Published:** 1907-03-23

**Authors:** 


### THE USE OF THE OPSONIC INDEX IN THE DIAGNOSIS OF TUBERCULOSIS.

At the Medico-Chirurgical Society of Edinburgh Drs. Struthers Stewart and Peel Ritchie communicated a note on the diagnosis of tuberculosis by means of the opsonic index. The normal opsonic index ranges from 0.8 to 1.2, and while any figure markedly below this should lead to a suspicion of tuberculosis, a single examination is insufficient for diagnostic purposes. The writers have utilised the observation that, while in non-tuberculous persons the injection of a small dose of tuberculin causes an immediate rise in the opsonic index, where tubercle is present a negative phase of variable intensity is first produced. The quantity of TR used was 1/500 milligramme in adults, or half that dose in children under 12 years of age, and the opsonic index was observed both the day before and the two days following the injection. By observation of a series of cases of known tuberculosis controlled by a series of non-tuberculous conditions, this method gave results in agreement with clinical methods in over 90 per cent. of cases, while a single observation without injection left a much larger margin of error.

